# Crystal structure of poly[di­aqua­(μ-2-carb­oxy­acetato-κ^3^ *O*,*O*′:*O*′′)(2-carb­oxy­acetato-κ*O*)di-μ-chlorido-dicobalt(II)]

**DOI:** 10.1107/S2056989015023269

**Published:** 2016-01-01

**Authors:** Yasmina Bouaoud, Zouaoui Setifi, Andrii Buvailo, Vadim A. Potaskalov, Hocine Merazig, Georges Dénés

**Affiliations:** a Unité de Recherche de Chimie de l’Environnement et Moléculaire Structurale (CHEMS), Université Constantine 1, Constantine 25000, Algeria; b Laboratoire de Chimie, Ingénierie Moléculaire et Nanostructures (LCIMN), Université Ferhat Abbas Sétif 1, Sétif 19000, Algeria; c National Taras Shevchenko University of Kyiv, Department of Chemistry, Volodymyrska str. 64, 01601 Kiev, Ukraine; d SciMax LLC, 2 Marshala Yakubovskogo str. 03191, Kyiv, Ukraine; e Department of General and Inorganic Chemistry, National Technical University of Ukraine, ‘Kyiv Polytechnic Institute’, 37 Prospect Peremogy, 03056 Kiev, Ukraine; f Laboratory of Solid State Chemistry and Mössbauer Spectroscopy, Laboratories for Inorganic Materials, Department of Chemistry and Biochemistry, Concordia University, Montréal, Québec, H3G 1M8, Canada

**Keywords:** crystal structure, malonate, cobalt, coordination polymer

## Abstract

The asymmetric unit of the title polymer, [Co_2_(C_3_H_3_O_4_)_2_Cl_2_(H_2_O)_2_]_*n*_, comprises one Co^II^ atom, one water mol­ecule, one singly deprotonated malonic acid mol­ecule (HMal^−^; systematic name 2-carb­oxy­acetate) and one Cl^−^ anion. The Co^II^ atom is octa­hedrally coordinated by the O atom of a water mol­ecule, by one terminally bound carboxyl­ate O atom of an HMal^−^ anion and by two O atoms of a chelating HMal^−^ anion, as well as by two Cl^−^ anions. The Cl^−^ anions bridge two Co^II^ atoms, forming a centrosymmetric Co_2_Cl_2_ core. Each malonate ligand is involved in the formation of six-membered chelate rings involving one Co^II^ atom of the dinuclear unit and at the same time is coordinating to another Co^II^ atom of a neighbouring dinuclear unit in a bridging mode. The combination of chelating and bridging coordination modes leads to the formation of a two-dimensional coordination polymer extending parallel to (001). Within a layer, O—H_water_⋯Cl and O—H_water_⋯O hydrogen bonds are present. Adjacent layers are linked through O—H⋯O=C hydrogen bonds involving the carb­oxy­lic acid OH and carbonyl groups.

## Abstract 2

In the title coordination polymer, [Co(C_3_H_3_O_4_)Cl(H_2_O)]_*n*_, the sixfold coordination environment of the Co^II^ atom consists of two O atoms from a chelating hydrogen malonate anion (HMal^−^), one O atom originating from a μ_2_-bridging malonate ligand (HMal^−^), one O atom from a water mol­ecule and two μ_2_-bridging Cl^−^ atoms, connecting neighbouring Co_2_Cl_4_ motifs into a two-dimensional polymer extending parallel to (001). Inter­layer O—H⋯O hydrogen bonds link the layers into a three-dimensional network.

## Chemical context   

Complexes with paramagnetic metal ions and extended structures are inter­esting due to their potential applications in mol­ecular magnetism (Moroz *et al.*, 2012 ▸; Pavlishchuk *et al.*, 2010 ▸, 2011 ▸; Yuste *et al.*, 2009 ▸). Malonic acid exhibits both chelating and bridging modes of coordination and is an efficient ligand for achieving two- or three-dimensional polymeric structures (Delgado *et al.*, 2004 ▸). In the present communication we report on the structure of a two-dimensional coord­ination polymer, [Co(C_3_H_3_O_4_)Cl(H_2_O)]_*n*_, containing both chelating and bridging functions of singly deprotonated malonic acid ligands.

## Structural commentary   

The structure of the title compound is characterized by the presence of a two-dimensional coordination polymer extending parallel to (001). The monomeric fragment can be described as being composed of a centrosymmetric binuclear Co_2_Cl_4_ motif with the Co^II^ atoms having an overall distorted octa­hedral environment. The two octa­hedra are fused together *via* two bridging Cl atoms with Co—Cl bond lengths of 2.4312 (12) and 2.4657 (16) Å. 
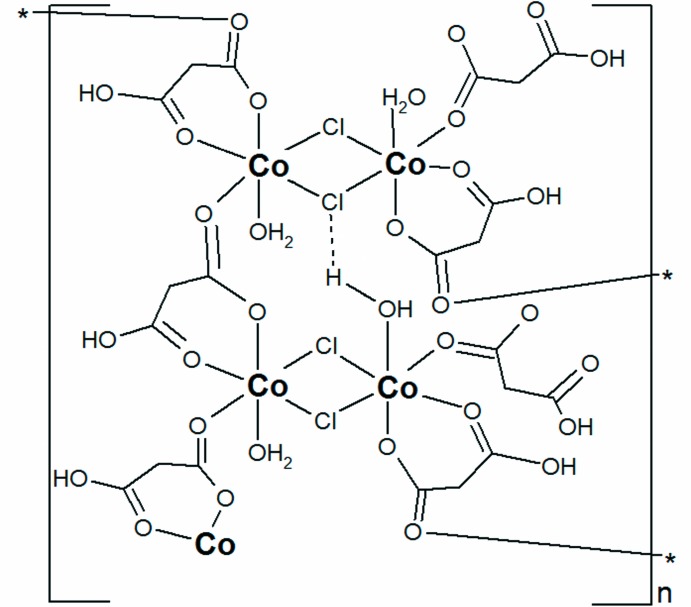


**Figure 1:**
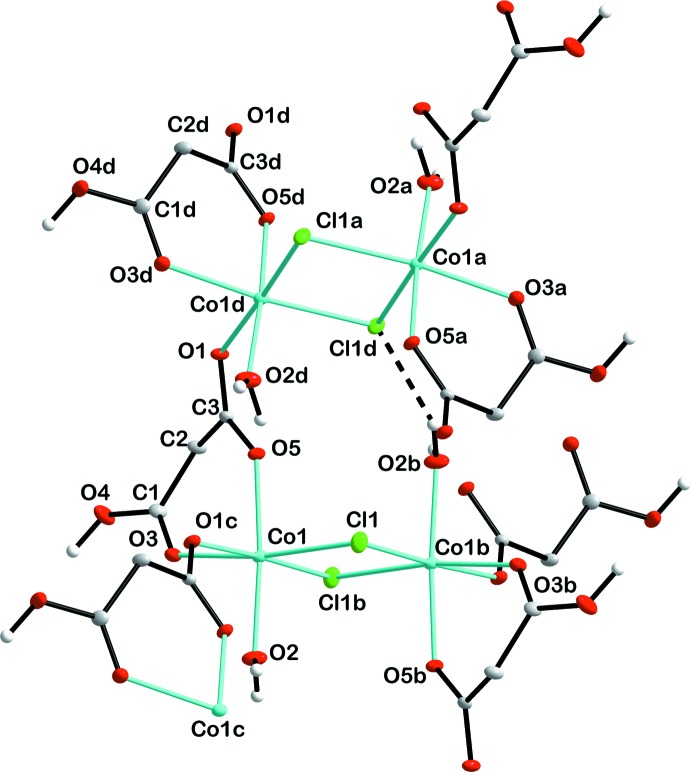
A fragment of the title coordination polymer, showing the atom labelling. All H atoms, except those of hy­droxy groups, have been omitted for clarity. Displacement ellipsoids are drawn at the 30% probability level. The intra­layer O—H⋯Cl hydrogen bonds are shown as dashed lines. [Symmetry codes: (a) 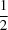 + *x*, 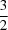 − *y*, 1 − *z*; (b) 1 − *x*, 1 − *y*, 1 − *z*; (c) 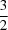 − *x*, −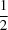 + *y*, *z*; (d) 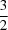 − *x*, 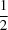 + *y*, *z*.]

In the octa­hedron, the Cl^−^ atoms occupy equatorial positions, the other two equatorial positions being defined by the carboxyl­ate O atom of a bridging hydrogenmalonate anion (HMal^−^) and one O atom of a chelating HMal^−^ anion, while one water O atom and the other O atom of the chelating HMal^−^ anion are in axial positions (Fig. 1 ▸). The corresponding Co—O_malonate_ bond lengths range from 2.051 (3) to 2.165 (3) Å which is similar to other structures containing this ligand in chelating and bridging modes (Delgado *et al.*, 2004 ▸). The Co—O_water_ bond has a length of 2.046 (3) Å. The C—O bond lengths in the carb­oxy­lic group differ significantly [1.225 (2) and 1.306 (4) Å] while those in the carboxyl­ate group [1.258 (4) and 1.267 (4) Å] are more or less the same, which is typical for this functional group (Wörl *et al.*, 2005*a* ▸,*b* ▸).

## Supra­molecular features   

**Figure 2:**
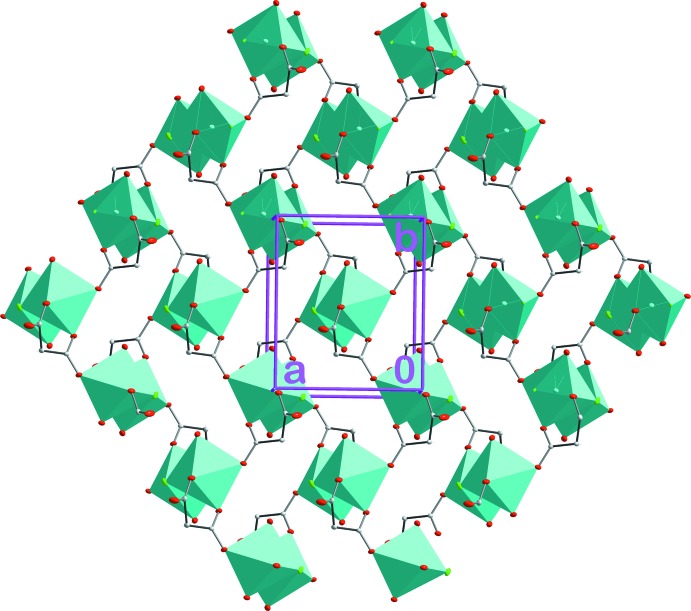
A view of the polymeric coordination layer in the crystal of the title compound, extending parallel to (001).

**Figure 3:**
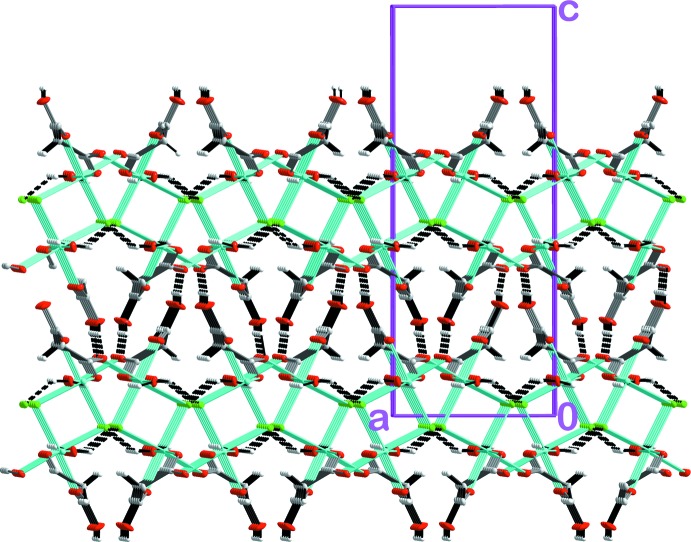
A view along [010] of the crystal packing of the title compound showing the inter- and intra­layer hydrogen-bonding system (dashed lines).

The distribution of the dinuclear units within a coordination layer follows a chess-like pattern whereby each dinuclear coordination node is inter­connected with each other through four bridging HMal^−^ ligands (Fig. 2 ▸). The binuclear coordin­ation nodes are additionally connected *via* intra­layer O—H_water_⋯Cl and O—H_water_⋯O hydrogen bonds (Table 1 ▸ and Fig. 3 ▸). Adjacent layers are linked along [001] *via* inter­layer O—H⋯O=C hydrogen bonds involving two HMal^−^ ligands (Table 1 ▸ and Fig. 3 ▸).

## Database survey   

A search of the Cambridge Structural Database (Groom & Allen, 2014 ▸) revealed a number of coordination polymeric structures containing cobalt(II) malonate moieties in different coordination modes. While the most typical coordination mode of malonate ligands in polymeric structures appears to be a μ_3_-bridging mode of the fully deprotonated acid involving all four oxygen atoms (usually two of them forming a chelating ring with one Co^II^ atom) (Delgado *et al.*, 2004 ▸; Xue *et al.*, 2003 ▸; Lightfoot & Snedden, 1999 ▸; Walter-Levy *et al.*, 1973 ▸; Zheng & Xie, 2004 ▸; Montney *et al.*, 2008 ▸; Fu *et al.*, 2006 ▸; Djeghri *et al.*, 2006 ▸), there are also cases of less-common coordination modes in polymeric structures such as a μ_2_-bridging mode of the fully deprotonated ligand connecting two metal atoms (Gil de Muro *et al.*, 1999 ▸; Pérez-Yáñez *et al.*, 2009 ▸; Jin & Chen, 2007 ▸). Much less common in coordination polymers is a mono-deprotonated state of malonic acid (Adarsh *et al.*, 2010 ▸), while there are also few examples of non-polymeric coordination compounds (Walter-Levy *et al.*, 1973 ▸; Clarkson *et al.*, 2001 ▸; Wang *et al.*, 2005 ▸).

## Synthesis and crystallization   

The title compound was synthesized by heating together 0.104 g (1 mmol) malonic acid dissolved in 15 ml of propanol and 0.238 g (1 mmol) of CoCl_2_·6H_2_O dissolved in 5 ml of water. Violet crystals suitable for X-ray analysis were isolated after two weeks by slow evaporation of the solvent from the resulting mixture. Crystals were washed with small amounts of propanol and dried in air yielding 0.071 g (36%) of the title compound.

## Refinement   

Crystal data, data collection and structure refinement details are summarized in Table 2 ▸. H atoms bound to O atoms were located from a difference-Fourier map and constrained to ride on their parent atoms, with *U*_iso_(H) = 1.5 *U*_eq_(O). All C-bound H atoms were positioned geometrically and were also constrained to ride on their parent atoms, with C—H = 0.97 Å, and *U*_iso_(H) = 1.2*U*_eq_(C).

## Supplementary Material

Crystal structure: contains datablock(s) I. DOI: 10.1107/S2056989015023269/wm5235sup1.cif

Structure factors: contains datablock(s) I. DOI: 10.1107/S2056989015023269/wm5235Isup2.hkl

CCDC reference: 1440440

Additional supporting information:  crystallographic information; 3D view; checkCIF report

## Figures and Tables

**Table 1:** Hydrogen-bond geometry (Å, °)

*D*—H⋯*A*	*D*—H	H⋯*A*	*D*⋯*A*	*D*—H⋯*A*
O2—H1*O*2⋯O5^i^	0.93	1.94	2.689 (4)	136
O2—H2*O*2⋯Cl1^ii^	0.92	2.32	3.135 (3)	147
O4—H1*O*4⋯O1^iii^	0.97	1.67	2.629 (4)	169

Symmetry codes: (i) 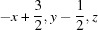; (ii) 
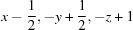
; (iii) 
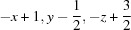
.

**Table 2:** Experimental details

Crystal data	
Chemical formula	[Co_2_(C_3_H_3_O_4_)_2_Cl_2_(H_2_O)_2_]
*M*_r_	430.90
Crystal system, space group	Orthorhombic, *P* *b* *c* *a*
Temperature (K)	296
*a*, *b*, *c* (Å)	7.568 (5), 8.879 (5), 19.168 (5)
*V* (Å^3^)	1288.0 (12)
*Z*	4
Radiation type	Mo *K*α
μ (mm^−1^)	3.04
Crystal size (mm)	0.20 × 0.14 × 0.07

Data collection	
Diffractometer	Nonius KappaCCD
Absorption correction	Multi-scan (*SADABS*; Bruker, 2004 ▸)
*T*_min_, *T*_max_	0.632, 0.820
No. of measured, independent and observed [*I* > 2σ(*I*)] reflections	6888, 1875, 1400
*R*_int_	0.055
(sin θ/λ)_max_ (Å^−1^)	0.704

Refinement	
*R*[*F* ^2^ > 2σ(*F* ^2^)], *wR*(*F* ^2^), *S*	0.046, 0.116, 1.05
No. of reflections	1875
No. of parameters	91
H-atom treatment	H-atom parameters constrained
Δρ_max_, Δρ_min_ (e Å^−3^)	1.05, −1.00

Computer programs: *COLLECT* (Nonius, 2000 ▸), *DENZO*/*SCALEPACK* (Otwinowski & Minor, 1997 ▸), *SHELXS97* and *SHELXL97* (Sheldrick, 2008 ▸) and *DIAMOND* (Brandenburg, 2010 ▸).

## supplementary crystallographic information

### Crystal data

**Table:**

[Co_2_(C_3_H_3_O_4_)_2_Cl_2_(H_2_O)_2_]	*F*(000) = 856
*M**_r_* = 430.90	*D*_x_ = 2.222 Mg m^−^^3^
Orthorhombic, *P**b**c**a*	Mo *K*α radiation, λ = 0.71073 Å
Hall symbol: -P 2ac 2ab	Cell parameters from 1003 reflections
*a* = 7.568 (5) Å	θ = 3.4–27.6°
*b* = 8.879 (5) Å	µ = 3.04 mm^−^^1^
*c* = 19.168 (5) Å	*T* = 296 K
*V* = 1288.0 (12) Å^3^	Block, violet
*Z* = 4	0.20 × 0.14 × 0.07 mm

### Data collection

**Table:**

Nonius KappaCCD diffractometer	1875 independent reflections
Radiation source: fine-focus sealed tube	1400 reflections with *I* > 2σ(*I*)
Horizontally mounted graphite crystal monochromator	*R*_int_ = 0.055
Detector resolution: 9 pixels mm^-1^	θ_max_ = 30.0°, θ_min_ = 3.4°
φ scans and ω scans with κ offset	*h* = −10→10
Absorption correction: multi-scan (*SADABS*; Bruker, 2004)	*k* = −12→12
*T*_min_ = 0.632, *T*_max_ = 0.820	*l* = −24→26
6888 measured reflections	

### Refinement

**Table:**

Refinement on *F*^2^	Primary atom site location: structure-invariant direct methods
Least-squares matrix: full	Secondary atom site location: difference Fourier map
*R*[*F*^2^ > 2σ(*F*^2^)] = 0.046	Hydrogen site location: inferred from neighbouring sites
*wR*(*F*^2^) = 0.116	H-atom parameters constrained
*S* = 1.05	*w* = 1/[σ^2^(*F*_o_^2^) + (0.0552*P*)^2^ + 0.9469*P*] where *P* = (*F*_o_^2^ + 2*F*_c_^2^)/3
1875 reflections	(Δ/σ)_max_ < 0.001
91 parameters	Δρ_max_ = 1.05 e Å^−^^3^
0 restraints	Δρ_min_ = −1.00 e Å^−^^3^

### Special details

**Table:**

Experimental. The O—H H atoms were located from the difference Fourier map but constrained to ride it's parent atom, with *U*_iso_ = 1.5 *U*_eq_(parent atom). Other H atoms were positioned geometrically and were also constrained to ride on their parent atoms, with C—H = 0.97 Å, and *U*_iso_ = 1.2 *U*_eq_(parent atom).
Geometry. All e.s.d.'s (except the e.s.d. in the dihedral angle between two l.s. planes) are estimated using the full covariance matrix. The cell e.s.d.'s are taken into account individually in the estimation of e.s.d.'s in distances, angles and torsion angles; correlations between e.s.d.'s in cell parameters are only used when they are defined by crystal symmetry. An approximate (isotropic) treatment of cell e.s.d.'s is used for estimating e.s.d.'s involving l.s. planes.
Refinement. Refinement of *F*^2^ against ALL reflections. The weighted *R*-factor *wR* and goodness of fit *S* are based on *F*^2^, conventional *R*-factors *R* are based on *F*, with *F* set to zero for negative *F*^2^. The threshold expression of *F*^2^ > σ(*F*^2^) is used only for calculating *R*-factors(gt) *etc*. and is not relevant to the choice of reflections for refinement. *R*-factors based on *F*^2^ are statistically about twice as large as those based on *F*, and *R*- factors based on ALL data will be even larger.

### Fractional atomic coordinates and isotropic or equivalent isotropic displacement parameters (Å^2^)

**Table:**

	*x*	*y*	*z*	*U*_iso_*/*U*_eq_	
Co1	0.57087 (6)	0.47694 (6)	0.58530 (2)	0.01449 (15)	
Cl1	0.71412 (10)	0.46924 (11)	0.47183 (5)	0.0223 (2)	
C1	0.4104 (4)	0.6347 (4)	0.70892 (19)	0.0177 (7)	
C2	0.4303 (4)	0.7853 (4)	0.6731 (2)	0.0176 (7)	
H2A	0.3323	0.7991	0.6411	0.021*	
H2B	0.4237	0.8644	0.7079	0.021*	
C3	0.6012 (4)	0.8016 (4)	0.63323 (18)	0.0133 (7)	
O1	0.6877 (3)	0.9227 (3)	0.64044 (14)	0.0179 (5)	
O2	0.5004 (3)	0.2544 (3)	0.58516 (16)	0.0276 (6)	
H1O2	0.5966	0.1929	0.5746	0.041*	
H2O2	0.3970	0.2004	0.5857	0.041*	
O3	0.4575 (3)	0.5133 (3)	0.68488 (14)	0.0202 (6)	
O4	0.3363 (4)	0.6465 (3)	0.77023 (15)	0.0321 (7)	
H1O4	0.3361	0.5574	0.7994	0.048*	
O5	0.6515 (3)	0.6967 (3)	0.59383 (13)	0.0175 (5)	

### Atomic displacement parameters (Å^2^)

**Table:**

	*U*^11^	*U*^22^	*U*^33^	*U*^12^	*U*^13^	*U*^23^
Co1	0.0156 (2)	0.0112 (2)	0.0167 (3)	−0.00042 (16)	−0.00079 (17)	−0.00210 (19)
Cl1	0.0181 (4)	0.0284 (5)	0.0204 (4)	0.0089 (3)	0.0002 (3)	−0.0038 (4)
C1	0.0123 (13)	0.0175 (19)	0.0233 (19)	−0.0019 (12)	0.0039 (13)	0.0013 (15)
C2	0.0146 (13)	0.0125 (17)	0.0257 (19)	0.0004 (12)	0.0054 (13)	0.0009 (15)
C3	0.0148 (13)	0.0099 (16)	0.0152 (17)	0.0007 (11)	0.0005 (11)	0.0014 (13)
O1	0.0206 (11)	0.0110 (12)	0.0220 (13)	−0.0028 (9)	0.0063 (10)	−0.0034 (11)
O2	0.0171 (11)	0.0177 (15)	0.0481 (19)	−0.0021 (10)	−0.0059 (12)	−0.0016 (13)
O3	0.0274 (13)	0.0133 (13)	0.0198 (14)	−0.0018 (10)	0.0038 (10)	−0.0005 (11)
O4	0.0514 (17)	0.0192 (15)	0.0257 (15)	0.0046 (13)	0.0211 (14)	0.0029 (12)
O5	0.0213 (11)	0.0116 (12)	0.0196 (13)	−0.0021 (9)	0.0052 (10)	−0.0047 (10)

### Geometric parameters (Å, º)

**Table:**

Co1—O2	2.046 (3)	C2—C3	1.509 (4)
Co1—O5	2.051 (3)	C2—H2A	0.9700
Co1—O3	2.118 (3)	C2—H2B	0.9700
Co1—O1^i^	2.165 (3)	C3—O5	1.258 (4)
Co1—Cl1	2.4312 (12)	C3—O1	1.267 (4)
Co1—Cl1^ii^	2.4657 (16)	O1—Co1^iii^	2.165 (3)
Cl1—Co1^ii^	2.4657 (16)	O2—H1O2	0.9325
C1—O3	1.225 (5)	O2—H2O2	0.9180
C1—O4	1.306 (4)	O4—H1O4	0.9698
C1—C2	1.511 (5)		
			
O2—Co1—O5	174.98 (11)	O4—C1—C2	112.4 (3)
O2—Co1—O3	92.46 (11)	C3—C2—C1	113.6 (3)
O5—Co1—O3	84.46 (10)	C3—C2—H2A	108.8
O2—Co1—O1^i^	90.35 (10)	C1—C2—H2A	108.8
O5—Co1—O1^i^	85.50 (10)	C3—C2—H2B	108.8
O3—Co1—O1^i^	86.33 (10)	C1—C2—H2B	108.8
O2—Co1—Cl1	95.04 (9)	H2A—C2—H2B	107.7
O5—Co1—Cl1	88.02 (7)	O5—C3—O1	122.5 (3)
O3—Co1—Cl1	172.49 (8)	O5—C3—C2	119.5 (3)
O1^i^—Co1—Cl1	93.10 (8)	O1—C3—C2	118.0 (3)
O2—Co1—Cl1^ii^	87.62 (8)	C3—O1—Co1^iii^	124.9 (2)
O5—Co1—Cl1^ii^	96.38 (8)	Co1—O2—H1O2	111.3
O3—Co1—Cl1^ii^	90.93 (8)	Co1—O2—H2O2	136.6
O1^i^—Co1—Cl1^ii^	176.52 (8)	H1O2—O2—H2O2	111.2
Cl1—Co1—Cl1^ii^	89.89 (4)	C1—O3—Co1	126.2 (3)
Co1—Cl1—Co1^ii^	90.11 (4)	C1—O4—H1O4	117.0
O3—C1—O4	122.3 (4)	C3—O5—Co1	131.5 (2)
O3—C1—C2	125.3 (3)		
			
O2—Co1—Cl1—Co1^ii^	87.60 (8)	C2—C1—O3—Co1	−2.5 (5)
O5—Co1—Cl1—Co1^ii^	−96.39 (8)	O2—Co1—O3—C1	−158.3 (3)
O1^i^—Co1—Cl1—Co1^ii^	178.22 (8)	O5—Co1—O3—C1	25.7 (3)
Cl1^ii^—Co1—Cl1—Co1^ii^	0.0	O1^i^—Co1—O3—C1	111.5 (3)
O3—C1—C2—C3	−38.3 (5)	Cl1^ii^—Co1—O3—C1	−70.6 (3)
O4—C1—C2—C3	141.5 (3)	O1—C3—O5—Co1	166.5 (2)
C1—C2—C3—O5	46.5 (5)	C2—C3—O5—Co1	−14.4 (5)
C1—C2—C3—O1	−134.3 (4)	O3—Co1—O5—C3	−17.1 (3)
O5—C3—O1—Co1^iii^	2.2 (5)	O1^i^—Co1—O5—C3	−103.9 (3)
C2—C3—O1—Co1^iii^	−176.9 (2)	Cl1—Co1—O5—C3	162.9 (3)
O4—C1—O3—Co1	177.8 (2)	Cl1^ii^—Co1—O5—C3	73.2 (3)

Symmetry codes: (i) −*x*+3/2, *y*−1/2, *z*; (ii) −*x*+1, −*y*+1, −*z*+1; (iii) −*x*+3/2, *y*+1/2, *z*.

### Hydrogen-bond geometry (Å, º)

**Table:**

*D*—H···*A*	*D*—H	H···*A*	*D*···*A*	*D*—H···*A*
O2—H1*O*2···O5^i^	0.93	1.94	2.689 (4)	136
O2—H2*O*2···Cl1^iv^	0.92	2.32	3.135 (3)	147
O4—H1*O*4···O1^v^	0.97	1.67	2.629 (4)	169

Symmetry codes: (i) −*x*+3/2, *y*−1/2, *z*; (iv) *x*−1/2, −*y*+1/2, −*z*+1; (v) −*x*+1, *y*−1/2, −*z*+3/2.
